# Stereolithography 3D Printing of a Heat Exchanger for Advanced Temperature Control in Wire Myography

**DOI:** 10.3390/polym14030471

**Published:** 2022-01-25

**Authors:** Leonardo Kelava, Ivan Ivić, Eszter Pakai, Kata Fekete, Peter Maroti, Roland Told, Zoltan Ujfalusi, Andras Garami

**Affiliations:** 1 Department of Thermophysiology, Institute for Translational Medicine, Medical School, University of Pecs, H-7624 Pecs, Hungary; leonardo.kelava@aok.pte.hu (L.K.); ivic.ivan@gmail.com (I.I.); eszter.pakai@aok.pte.hu (E.P.); kata.fekete@aok.pte.hu (K.F.); 2 Medical Simulation Education Center, Medical School, University of Pecs, H-7624 Pecs, Hungary; marotipeter8@gmail.com (P.M.); told.roland@pte.hu (R.T.); 3 3D Printing and Visualization Center, University of Pecs, H-7624 Pecs, Hungary; 4 Department of Biophysics, Medical School, University of Pecs, H-7624 Pecs, Hungary; zoltan.ujfalusi@aok.pte.hu

**Keywords:** 3D printing, SLA, resin, mechanical characterization, thermal conduction, heat exchange, wire myograph, thermoregulation, thermophysiology

## Abstract

We report the additive manufacturing of a heat-exchange device that can be used as a cooling accessory in a wire myograph. Wire myography is used for measuring vasomotor responses in small resistance arteries; however, the commercially available devices are not capable of active cooling. Here, we critically evaluated a transparent resin material, in terms of mechanical, structural, and thermal behavior. Tensile strength tests (67.66 ± 1.31 MPa), Charpy impact strength test (20.70 ± 2.30 kJ/m^2^), and Shore D hardness measurements (83.0 ± 0.47) underlined the mechanical stability of the material, supported by digital microscopy, which revealed a glass-like structure. Differential scanning calorimetry with thermogravimetry analysis and thermal conductivity measurements showed heat stability until ~250 °C and effective heat insulation. The 3D-printed heat exchanger was tested in thermophysiology experiments measuring the vasomotor responses of rat tail arteries at different temperatures (13, 16, and 36 °C). The heat-exchange device was successfully used as an accessory of the wire myograph system to cool down the experimental chambers and steadily maintain the targeted temperatures. We observed temperature-dependent differences in the vasoconstriction induced by phenylephrine and KCl. In conclusion, the transparent resin material can be used in additive manufacturing of heat-exchange devices for biomedical research, such as wire myography. Our animal experiments underline the importance of temperature-dependent physiological mechanisms, which should be further studied to understand the background of the thermal changes and their consequences.

## 1. Introduction

Additive manufacturing is an increasingly applied technology in medicine and biomedical science [1,2], while it is also a cost-effective, reliable, and user-friendly tool for the creation of laboratory equipment [3]. Heat exchangers are widely used in thermal investigations both in engineering and in life sciences, and additive manufacturing has become an essential tool in the development and final production of these devices [4,5]. Besides metals and ceramics, which both have favorable thermal characteristics, polymers are also used to produce heat exchangers. In a previous study, Arie et al. used HDPE (high-density polyethylene) and PLA (polylactic acid) for additively manufacturing a polymer heat-exchanging device with excellent heat transfer performance [6]; however, the SLS (selective laser sintering) technology was also successfully applied to produce a polymer heat exchanger with good thermal performance [7]. In a recent study, the use of a metal (aluminum) and polymer composite (ABS, acrylonitrile butadiene styrene) was highlighted [8], while another research group used resin-based composites for thermal investigation [9]. Furthermore, to increase stiffness and hardness, dos Santos et al. developed a composite of photocurable epoxy-acrylate resin and multiwalled carbon nanotubes [10]. Surprisingly, to our knowledge, there is no data available in the scientific literature about the use of resins and desktop stereolithography (SLA) technology for heat exchanger development and about fabrication of heat exchangers with standard, transparent, one-component resins, despite their broad availability and versatility. Previously, SLA 3D printing technology has been utilized only for the optimization of microchannel cooling configurations [11,12], but not for the development of a cooling device for biomedical research purposes, at which we aimed, for the first time to our knowledge, in the present study. Furthermore, the detailed thermal characteristics of the printouts of transparent resins, i.e., their differential scanning calorimetry with thermogravimetry analysis (DSC-TGA), have remained unknown, even though the DSC-TGA is an essential tool for efficiently and accurately mapping the thermal properties of heat-exchanging materials, thereby providing additional information about the thermal performance of the device to the users. Testing of the mechanical and thermal properties of 3D-printed parts is of high importance, because it can provide valuable information about the fusion of layers during printing, thus, about part quality and strength [13], as well as about the relations of the thermal and the fatigue parameters of the 3D-printed material under static and cyclic loading, including the self-heating phenomenon [14].

Efficient heat exchangers are particularly important for thermophysiology studies, in which body or organ responses at different temperatures are measured. In mammals, blood vessels are integral part of the thermoregulation system and are biologically responsive to changes in temperature. In the skin, upon cold exposure they promote heat conservation through vasoconstriction, whereas, upon exposure to warmth, they facilitate heat dissipation through vasodilation. Via their thermal responses, peripheral (e.g., cutaneous) blood vessels also affect systemic blood pressure, tissue blood flow, and the production of biologically active molecules [15]. Temperature fluctuations can be particularly pronounced in the skin, especially in small mammals (e.g., mice and rats). For example, when mice were exposed to severe cold (8 °C for 180 min), their skin and deep body temperatures dropped from 31 to 8 °C and from 37 to 13 °C, respectively [16]; still, they all recovered from the hypothermia after cessation of the cold exposure. Compared to small rodents, the larger body mass of humans ensures better heat conservation; nevertheless, in extreme situations, similarly low body temperatures as in the mouse experiments can also occur in humans. For example, a deep body temperature as low as 13.7 °C was recorded in a human subject in a skiing accident. It is notable that the patient survived with minimal consequences and no brain damage, which could be due to the rapid cooling of the brain leading to decreased metabolic rate, thereby to minimal ischemic damage [17]. Although it was shown that vasomotor responses to some substances differ at different temperatures [18], it is a challenge to perform the direct measurement of the vasomotor responses of isolated arteries in the cold, because of the lack of proper experimental methods possessing tight temperature control in cold environments. Wire myography was developed in the 1970s as a technique specifically designed to measure contractile forces of isolated vascular preparations [19]. At present, several manufacturers offer wire myographs with different technical specifications. It is a common feature that these instruments have integrated heaters, allowing to heat the baths containing the isolated vessels segment to temperatures that are above the room temperature, whereas none of them has the ability to actively cool the bath below the room temperature, thereby limiting their potential use for mimicking cold exposure, thus, to study vasomotor responses in the cold. Here, we present the development and the prototype of a unique heat exchanger as an accessory to the wire myograph, which is capable of tight temperature control of the bath in a wide range, also including its active cooling well below room temperature. Preliminary tests indicated that SLA 3D printing with a transparent resin could be used for additive manufacturing of the heat exchanger. We aimed to evaluate and characterize a commercially available resin for desktop SLA and to critically investigate its potential use in the fabrication of heat-exchange devices for ex vivo thermophysiology studies.

## 2. Materials and Methods

### 2.1. 3D Printing and Mechanical Testing

In order to fabricate a watertight system, SLA additive manufacturing technology was applied. For 3D printing, a Formlabs Form 2 SLA desktop printer was used (Formlabs Inc., Somerville, MA, USA), and the selected material was the transparent Clear Resin V4 (Formlabs Inc., Somerville, MA, USA). To fabricate the test specimens for mechanical testing and thermal conductivity, the printer has been set to the parameters shown in Table 1. The parameters were chosen based on the material data sheet of the resin and based on our previous experience with the fabrication of microfluidic systems.

#### 2.1.1. 3-Point Flexural Test

The 3-point bending tests were carried out by a Zwick/Roell Z100THW universal material tester (ZwickRoell, Ulm, Germany). The tests were conducted according to ISO 178:2010 with the fabricated specimen. According to the standard, the size of specimen was 4 mm × 10 mm × 80 mm. The pre-load was 0.1 MPa and the speed was set at 2 mm/min during the entire test. The support distance was 64 mm and the maximum deformation was 4.7%. The tests were performed 5 times.

#### 2.1.2. Tensile Test

The tensile tests were carried out with the same Zwick/Roell Z100THW tester (ZwickRoell, Ulm, Germany), supplemented with and extensometer. The tests were conducted according to ISO 527-1:2019 with the fabricated specimen. The type of specimen was A1 from ISO 527-2:2012 and the pre-load was 0.1 MPa. The speed was set at 1 mm/min during the determination of Young’s modulus, while it was 50 mm/min during the test. The number of test specimens was 5.

#### 2.1.3. Charpy Test

The Charpy impact tests were performed by a Zwick/Roell Hit50P instrument with 5 J pendulum (ZwickRoell, Ulm, Germany) according to the 179-1:2010 standard: the size of specimen was 4 mm × 10 mm × 80 mm; the hit impacted the edge; and the tests were repeated 5 times.

#### 2.1.4. Shore D Hardness

The Shore D hardness tester was a Zwick/Roell 3131/320154 device (ZwickRoell, Ulm, Germany). The tests were performed according to the 868:2003 standard. The instrument was on a stand and the specimen thickness was 5 mm. All measurements were carried out 5 times.

### 2.2. Thermal Conductivity

**Figure 1:**
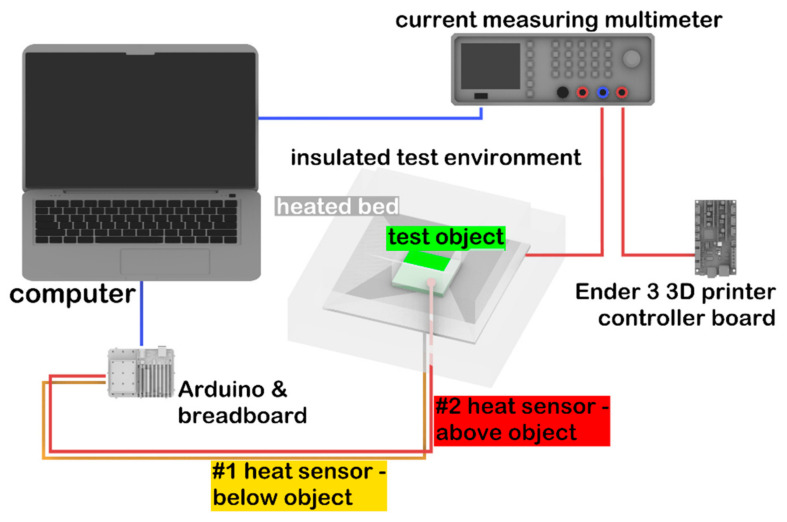
Schematic representation of the experimental setup used for thermal conductivity measurements.

Thermal conductivity measurements were performed on 3 test samples. In order to perform the measurement, an apparatus was built with the following elements (see also Figure 1):

- Arduino Uno (Arduino, Somerville, MA, USA) with 2 thermistors;
- Rohde@Schwarz HMC8012 Digital Multimeter (Rohde & Schwarz, München, Germany);
- Ender 3 3D printer controller (Creality, Shenzhen, China) for control of the heated bed;
- 24 V, 160 W heated bed (Creality, Shenzhen, China);
- 8-cm thick Energosystem ES-EPS-80-8 polystyrene (Thermotrading Ltd., Cegléd, Hungary) for insulation of the measured system from the environment;
- Thermal grease (model: 51634; Qoltec, Gliwice, Poland) for thermal conductive adhesion.

The heating plate was surrounded by polystyrene foam (*λ* = 0.039 W/mK) as an insulation, except for a 7 cm × 7 cm wide opening, through which the test specimens were placed inside. The size of the 3D-printed test specimen was 5 mm × 70 mm × 70 mm. For the calibration of the device, a glass plate was used, which had the same size as the sample (*λ*_ref_ = 1 W/mK). Thermal sensors were positioned on the top and the bottom of the test specimen, and then the sample was placed on the heating bed. Measurements were recorded by the Arduino Uno with a frequency of 0.2/s. The heat flow was determined by the electric energy consumption of the heating bed. The multimeter was serially connected to the heating bed and the Ender 3 printer controller. Power consumption as a function of time was recorded on a personal computer (Figure 1).

The measurements were performed in five stages in the case of both glass and resin samples: (i) 35 °C for 45 min; (ii) 37 °C for 35 min; (iii) 44 °C for 30 min; (iv) 49 °C for 25 min; and (v) 58 °C for 20 min. The duration of a full heating cycle was 155 min. During the entire measurement cycle the heating bed was completely surrounded with polystyrene (except the 7 cm × 7 cm opening); thus, heat conductivity could be determined by measuring the loss of the current (for details, see below). Further data processing was performed in the Origin software (OriginLab Corporation, Northampton, MA, USA). In each phase, after reaching the thermodynamic equilibrium, the difference between the two thermosensors was determined by averaging the difference data obtained in three full heating cycles.

Heat flow was calculated on the basis of the ratio of the maximum usable current and the current related to the actual heating consumed over the three cycles, and then the amount of heat loss was deducted from this value. After that, the following equation was used:

(1) $$\lambda =\left|\frac{\dot{Q}}{A\cdot \frac{T_{2}-T_{1}}{\delta }}\right|$$

where $\dot{Q}$—heat flow (W), *A*—cross section of the test specimen (m^2^), *T*_1_, *T*_2_—temperatures measured on both sides of the test specimen (°C), *δ*—thickness of the test specimen (m). The measured and calculated values were fitted to an exponential curve that was determined by the following equation:

(2) $$y=A\cdot e^{-\frac{x}{t}}+y_{0}$$

where *A*, *t*, *y*_0_ are constants and *x* marks the temperature.

The zero point correction factor of the device was determined by the following equation:

(3) $$k=\frac{\lambda_{reference}}{\lambda_{measured}}$$

where k is the correction factor and $\lambda_{reference}$ and $\lambda_{measured}\;$ are the heat conductions for the reference material as determined by the manufacturer and as measured in our experiments at 23 °C, respectively. The correction was made at the ambient temperature at which the measurement was performed (23 °C).

### 2.3. DSC-TGA

LabSys Evo device (Setaram Ltd., Caluire-et-Cuire, France) was used to perform these experiments. The calibration of the instrument was recently performed by the manufacturer. The measurements were carried out under 100 mL/min nitrogen atmosphere and the mass of each sample was set between 5.2–5.5 mg. The applied temperature range for the measurements started at 30 °C, ended at 750 °C, and was increased at a rate of 10 °C/min. Because of the high temperatures, uncovered Al_2_O_3_ crucibles (Setaram Ltd., Caluire-et-Cuire, France) with a volume of 100 µL were used. The rate of the sample’s spontaneous cooling was sufficient for the measurements; thus, the application of external cooler was not necessary. Data processing was performed using Origin 2021 software (OriginLab Corporation, Northampton, MA, USA).

### 2.4. Digital Microscopy

Images were taken of both the intact and broken surface of the specimens used in the Charpy test in order to evaluate the structure of the resin material with a König digital microscope (König Electronic GmbH, Reichelsheim, Germany) with 35× magnification.

### 2.5. Fabrication of the Heat-Exchange Device

**Figure 2:**
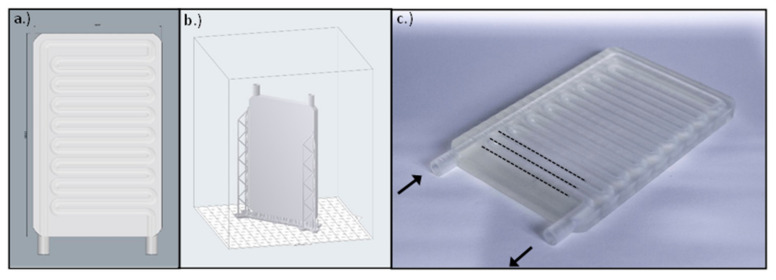
(**a**) The CAD model of the cooling plate created with Rhinoceros 6 CAD software. (**b**) The .stl model uploaded in the slicer software. (**c**) The manufactured cooling plate using a 3D printer. The arrows indicate the inflow and outflow pipes of the plate, and the dotted lines represent the parallel cooling pipes.

The instrument was designed using Rhinoceros 6 CAD (computer-aided design) software (Robert McNeel & Associates, Seattle, WA, USA). The size of the final product was 75 mm × 120 mm × 7 mm, the cooling pipes were 10.5 mm long with a 5 mm inner and 7 mm outer diameter and were interspaced from each other by 1 mm (Figure 2). The slicing process was performed by the PreForm slicing software (Formlabs Inc., Somerville, MA, USA). The layer height was set to 50 µm, and the device was in “Y” printing orientation on the printing bed. The overall printing time was 13 h and 15 min and it required a total volume of 46 mL resin. The post processing included bathing in isopropyl alcohol for 10 min and UV curing at 60 °C for 15 min.

### 2.6. Thermophysiology Experiments

#### 2.6.1. Animals

The thermophysiology experiments were performed in 22 Wistar rats. At the time of the experiments the rats aged 1–3 months and weighed 140–240 g. Animals were bred and kept at University of Pecs in standard plastic cages (model: 1290 D Eurostandard type III; Akronom Ltd. Budapest, Hungary) kept in a room with an ambient temperature maintained at 21–23 °C and humidity at 30–40%. The room was on a 12/12-h light/dark cycle (lights on at 5:00 a.m.). The rats had access to standard rodent chow and tap water ad libitum. All experiments were conducted under protocols approved by the Institutional Animal Use and Care Committee of the University of Pecs (registration no.: BA02/2000-6/2018, approved on 27 February 2018) and conformed to the guidelines from Directive 2010/63/EU of the European Parliament on the protection of animals used for scientific purposes.

#### 2.6.2. Experimental Procedures

The experiments were conducted in a multi wire myograph system (model DMT 610M; Danish Myo Technology A/S, Aarhus, Denmark). A heat-exchanger plate (developed in the present study) was placed on the top of the myo-interface unit underneath each of the four myograph chamber units. The heat-exchanger plates were connected to polyethylene peroxide-cured tubes, which were continuously perfused from a water tank. In the tank, the temperature of the water was tightly controlled with a heating and a cooling device (models GD120 and C1G, respectively; Grant Instruments Ltd., Cambridge, UK). Before each experiment, the calibration procedure of the myograph system was performed according to the manufacturer’s instructions, while the temperature of the bath containing Krebs solution (NaCl: 119 mM, KCl: 4.7 mM, KH_2_PO4: 1.2 mM, NaHCO_3_: 25 mM, Mg_2_SO_4_: 1.2 mM, CaCl_2_ × 2H_2_O: 1.6 mM, EDTA: 0.026 mM, and glucose: 11.1 mM) in the myograph chambers was maintained at 13, 16, or 37 °C. The ingredients of the Krebs solution were purchased from Sigma-Aldrich (St. Louis, MO, USA). The bath solution’s temperature was recorded with a 12-channel scanning thermocouple thermometer (Cole-Parmer, Vernon Hills, IL, USA) throughout the calibration procedure and the experiment. The thermocouples (Omega Engineering, Stamford, CT, USA) were calibrated, and then inserted in the bath solution in a way that they did not touch the walls of the chamber.

The isolation of the tail artery was performed as in our earlier study [20]. In brief, the rats were anesthetized by the intraperitoneal injection of a ketamine-xylazine cocktail (81.7 and 9.3 mg/kg, respectively). A midline incision was made on the top of the tail; then, the tail artery was isolated and cleaned from its connective tissue. The proximal and distal ends of the isolated artery were ligated and the vessel segment between the ligations was excised. After the removal of the artery, the animal was euthanized with an intraperitoneal injection of pentobarbital (100 mg/kg; Cave Santa Animale, Libourna, France). The excised artery was dissected into four 2-mm long segments. The segments were mounted in the myograph chambers using tungsten wires with 40 nm diameter and allowed to rest for 60 min; then, normalization was performed according the manufacturer’s instructions. After the normalization, the viability of the vessels was verified by the presence of vasoconstriction induced by 60 mM KCl. In the experiments, endothelium-dependent vasoconstriction was studied in response to increasing concentrations of phenylephrine (10^−8^ to 10^−4^ M; Sigma-Aldrich, St. Louis, MO, USA) added to the solution in 5 min intervals. After washout with Krebs solution, 60 mM KCl was used to evaluate the vessel’s viability at the end of the experiment.

### 2.7. Statistical Analysis

The statistical analysis of the data obtained with mechanical testing, calorimetry, and thermal conductivity measurements was performed with OriginPro 2018 software (OriginLab Corporation, Northampton, MA, USA). Results of the thermophysiology experiments were analyzed with the R software version 3.6.1 (R Development Core Team, Vienna, Austria). Since Levene’s test revealed unequal variances, Welch’s one-way ANOVA with post-hoc Games–Howell test was used to compare responses across different temperatures. Vasomotor responses to 60 and 90 mM KCl at 16 °C were compared with Student’s *t*-test.

## 3. Results

### 3.1. Mechanical Characterization

The results of the mechanical characterization underlined that the selected resin is hard, durable, and can withstand the planned experiments after fabrication of the heat-exchange device (Table 2). The average value of tensile strength was 67.66 ± 1.31 MPa, the flexural stress at standard deflection was measured as 78.82 ± 1.17 MPa, while the Charpy impact test resulted in 20.70 ± 2.30 kJ/m^2^. The shore D hardness was 83.00 ± 0.47.

In an earlier study, Epon 828^®^ resin, a commonly used epoxy polymer, exhibited tensile strength of 69 MPa and tensile Young’s modulus of 2750 MPa [21], which are slightly higher values than what we found for the resin used in present study (Table 2). A recent summary of the tensile strength and modulus of fused filament fabrication 3D-printed PLA parts indicated that, if the design parameters were similar (i.e., XYZ build orientation; 0–45° raster angle; 0.15–0.20 mm layer thickness), then tensile strength varied between 41 and 59 MPa and the tensile modulus ranged from 3130 to 3520 MPa [22]. In the same study, the authors fabricated PLA specimen with the same technology and similar design parameters (XYZ build orientation, 0.14 mm layer thickness), which had tensile strength of 31–58 MPa and tensile modulus of 2900–3130 MPa depending from the raster angle (0–90°) [22]. The tensile strength of the investigated material in the present study was higher, but it was also more elastic (Table 2).

### 3.2. Thermal Conductivity of the Resin Material

**Figure 3:**
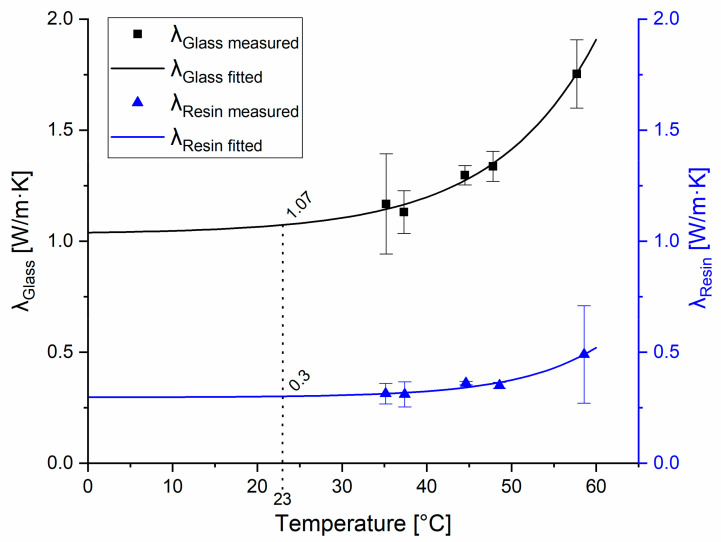
Measured and fitted heat conduction parameters of the tested materials. Heat conduction of the reference glass material is represented by the black curve; the squares show the average values with standard deviations measured at the indicated temperatures. Heat conduction parameters of the resin material are represented by the blue curve; triangles show the measured average values with standard deviations measured at the indicated temperatures.

The thermal conductivity measurements were performed on the transparent resin. Glass was used as a reference material. To the measured data points of resin, which were 0.314 W/mK at 35 °C, 0.311 W/mK at 37 °C, 0.361 W/mK at 44 °C, 0.351 W/mK at 49 °C, and 0.490 W/mK at 58 °C, a curve was fitted, and then the results were extrapolated based on the previously determined correction factor (k=0.931). After the calibration of the setup, the heat conduction parameters were measured at 35, 37, 44, 49, and 58 °C, which resulted in heat conduction of 0.314, 0.311, 0.361, 0.351, and 0.490 W/mK, respectively (Figure 3).

The corrected average of heat conduction for the tested resin material was 0.280 W/mK, which was considered constant at all temperatures. To our knowledge, this is the first study that reports the thermal conductivity of SLA 3D-printed clear resin (λ_clear resin_ = 0.280 W/mK), which is similar to the thermal conductivity of epoxy resin (λ_epoxy resin_ = 0.202 W/mK) as determined earlier by other authors [23].

### 3.3. Thermal Characterization of the Transparent Resin

**Figure 4:**
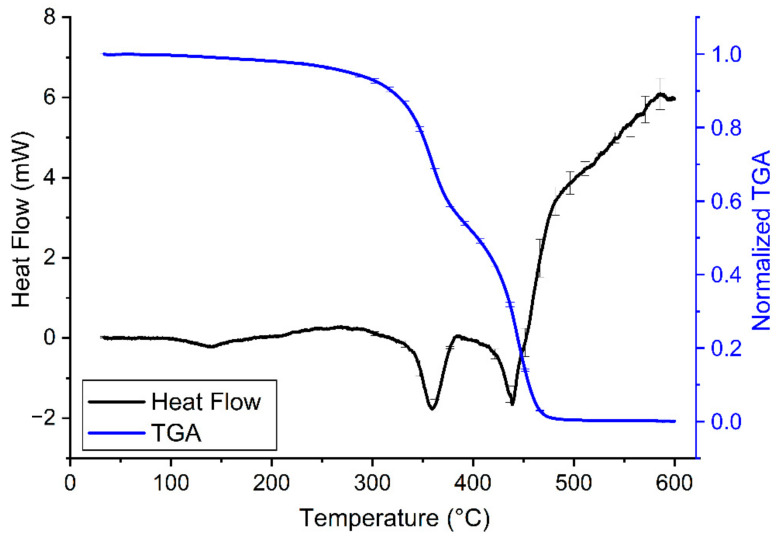
Heating curves of clear resin V4 (30–600 °C, black line) combined with TGA of the samples (30–600 °C, blue line) on a common temperature scale (i.e., abscissa) at a heating rate of 10 °C/min. On the heating curves, the endothermic process (i.e., when the sample melts) causes a drop in heat flow. A decrease in normalized TGA indicates loss of sample mass. The error bars show the standard error of means from at least 3 independent measurements.

The characterization of the thermal properties of the transparent resin samples revealed a relatively high heat stability of the printouts (Figure 4 and Table 3). According to the manufacturer, there is no significant change in the thermal properties of these samples until 250 °C, which has now also been confirmed with the results of our DSC-TGA (Figure 4). It should be noted, however, that a slight gradual loss of sample mass was present already from the beginning of the heating of the sample. The same tendency could be observed with ABS samples, though, in that case, the gradual decrease in sample mass started at higher temperatures [24]. Interestingly the TGA curve of the tested clear resin (Figure 4) is very similar to that of PLA shown in a previous study [24]. However, while PLA and other PLA-based composites usually have a melting temperature of approx. 150-170 °C [25], the clear resin stayed solid at that temperature and even far above (Table 3). Of note, other photopolymers, such as thermally cured structural self-adhesive tapes have much less thermal stability [26]. Such good thermal stability as the one observed in case of the SLA 3D-printed resin in the present study, can be achieved in case of several polymer materials only by the addition of nanoparticles or cellulose nanofibers to their structure, which can enhance various properties, including thermal stability [27,28]. Our research group has previously examined the thermal properties of 3D printouts from many composites, such as PLA, ABS, polyamide, high impact polystyrene, and polyethylene terephthalate glycol (data not shown) and none of these materials had the glass transition (Tg) and melting phases at such high temperatures as the transparent resin. However, the appearance of the fast phase of mass decrease at around 300 °C suggests that resin quickly destabilizes and loses its previous rigid structure above this temperature (Figure 4).

The quick phase-change is confirmed by the synchronicity of the prompt decrease in sample mass with the endothermic reaction (i.e., the melting of the sample). This indicates that pyrolysis and the melting of the sample are parallel. Based on our TGA, at around 500 °C, the majority of the sample was pyrolyzed (Figure 4).

### 3.4. Digital Microscopy

**Figure 5:**
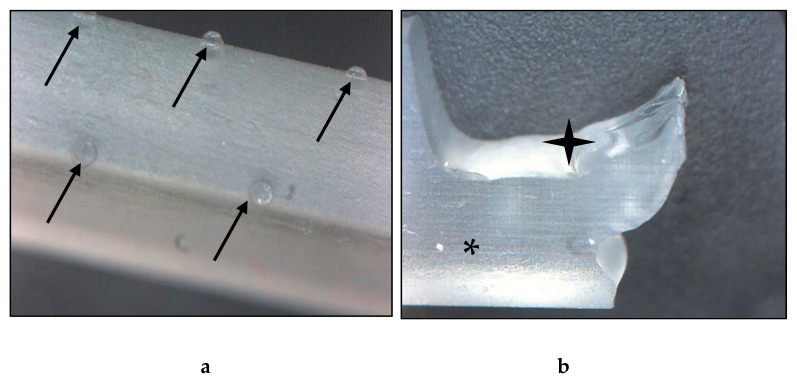
Images of the fabricated resin material taken with a digital microscope. (**a**) Intact test specimen, surface on the edge. The black arrows point to the protrusions, which are the remains of the raft. (**b**) Broken test specimen. The black star indicates the broken, the asterisk marks the intact surface, where the glassy-like structure can be observed. Magnification: 35×.

The images taken with the digital microscope revealed important information about the structure of the resin material. The fabricated material had an extremely smooth, glass-like surface, and a glassy breaking pattern. The layers were not clearly visible; they could not be separated visually with the used 35× magnification. The material was nearly transparent, therefore, ideal for the production of heat-exchange devices. On the intact surface, small bumps with 0.5 mm diameter could be observed. The raft, which is fundamental in this technology was connected on these protrusions, which must be considered during the 3D modeling and printing processes (Figure 5).

### 3.5. Thermophysiology Experiments

The application of the heat-exchange device effectively reduced and maintained the temperature of the bath solution in all chambers of the wire myograph system at 13 and 16 °C. There was no meaningful difference in the temperature among the chambers as indicated by the relatively small standard deviations of the mean temperature of the four chambers: 0.72 °C at 13 °C and 16 °C, while 0.36 °C at 36 °C. There seemed to be a negative correlation between the target temperature and the standard deviation of the mean chamber temperature, but it did not reach the level of significance (corr = −0.50, *p* = 0.08).

**Figure 6:**
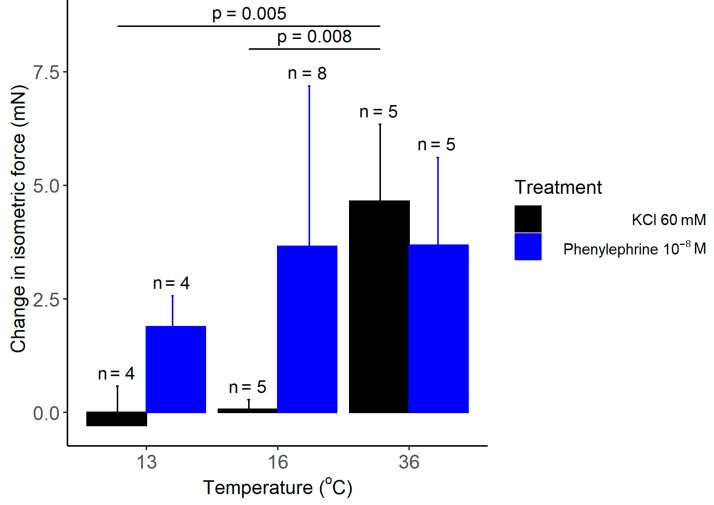
The change in the isometric force of isolated rat tail arteries after adding KCl or phenylephrine (doses indicated) to the bath solution maintained with the manufactured heat-exchanger plates at 13, 16, or 36 °C. Data are presented as mean ± standard deviation.

As expected from earlier studies [20], we registered a significant increase in the isometric force in response to administration of 60 mM KCl, when the bath solution of the isolated rat tail arteries was maintained at 36 °C (Figure 6). However, when the temperature of the bath solution was reduced to 13 or 16 °C with the newly developed heat-exchange device, administration of the same concentration of KCl had no meaningful effect on the isometric force. In order to exclude the possibility that the wire myograph system or the arteries are not functional at 13 or 16 °C, we used phenylephrine, which causes vasoconstriction via distinct mechanisms from KCl. When we administered phenylephrine (10^−8^ to 10^−4^ M) under the same experimental conditions, it caused a marked increase in the isometric force at all tested temperatures (Figure 6), thereby indicating that the attenuation of the KCl-induced vasoconstriction is not due to methodological reasons, but instead it results from the cold-induced suppression of the vasomotor response to KCl.

**Figure 7:**
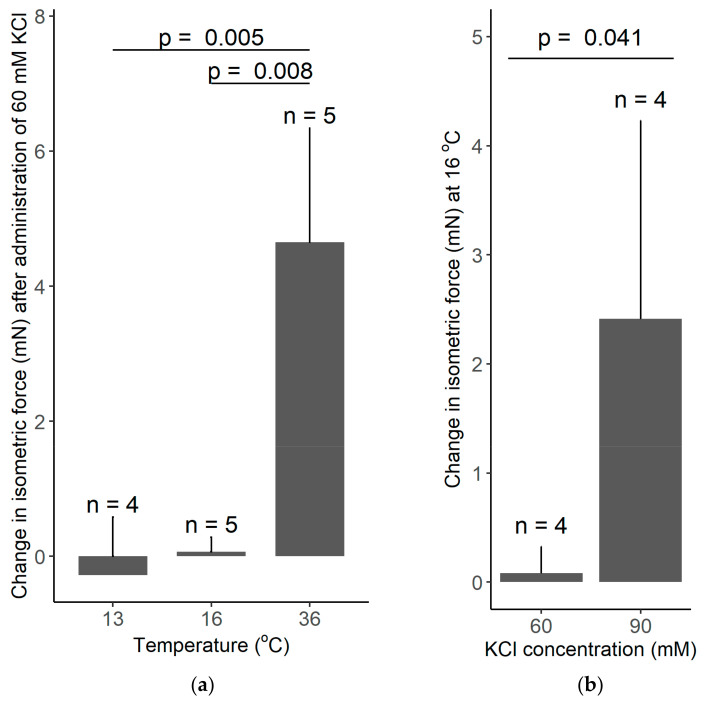
(**a**) The change in the isometric force of isolated rat tail arteries after adding 60 mM KCl to the bath solution maintained with the manufactured heat-exchanger plates at 13, 16, or 36 °C. Note that the KCl-induced vasoconstriction was abolished at low temperatures of 13 and 16 °C. (**b**) The change in the isometric force of isolated rat tail arteries after adding 60 or 90 mM KCl to the bath solution maintained at 16 °C. The higher concentration of KCl caused a significant vasoconstriction. Data are presented as mean ± standard deviation.

In our thermophysiology experiments, the recorded increase in the isometric force in response to 60 mM KCl was 4.65 mN at 36 °C, whereas it was practically absent at 13 and 16 °C (Figure 7a). Welch ANOVA showed a significant effect of temperature on the KCl-induced vasomotor response (*p* = 0.002). With Games–Howell post hoc test, we found a significant difference in the KCl-induced change in isometric force between 13 and 36 °C (*p* = 0.005), as well as, between 16 and 36 °C (*p* = 0.008), while there was no significant difference between 13 and 16 °C. However, when the concentration of KCl was increased from 60 to 90 mM, we found a significant (*p* = 0.041) increase in the isometric force of the tail artery at 16 °C (Figure 7b). The latter finding further supports the capability of the used experimental setup to record vasomotor responses in the cold; furthermore it underlies the existence of temperature-dependent differences in vascular responses.

**Figure 8:**
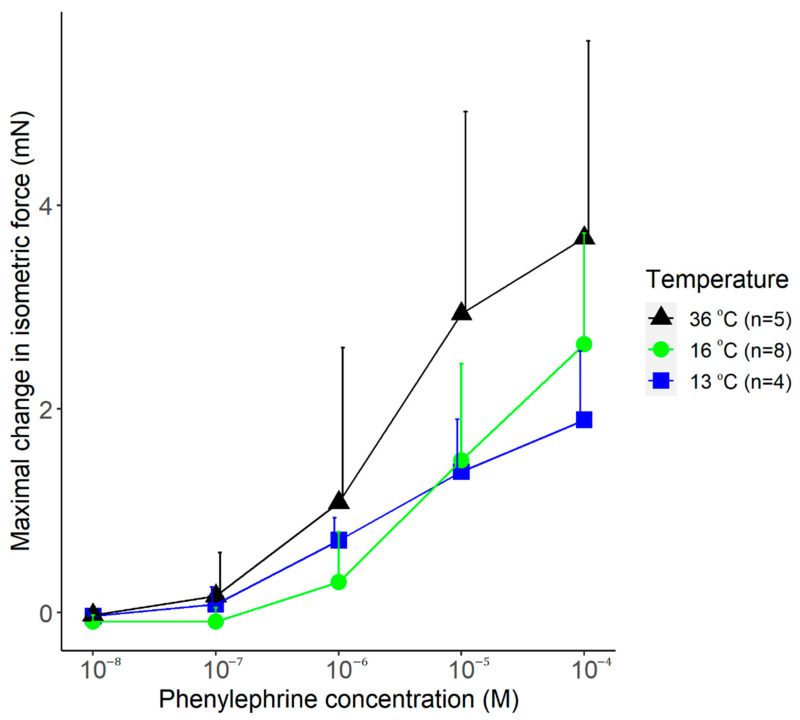
The maximal change in the isometric force of isolated rat tail arteries after adding phenylephrine (doses indicated) to the bath solution maintained with the manufactured heat-exchanger plates at 13, 16, or 36 °C. Data are presented as mean ± standard deviation.

The vasomotor response to increasing doses of phenylephrine was also studied at the different temperatures. Phenylephrine caused an increase in the isometric force of the arteries at all temperatures (viz., 13, 16, and 36 °C) with similar dynamics (Figure 8). The maximal vasomotor response tended to be smaller in the cold (at 13 and 16 °C) than at 36 °C, but the effect of temperature did not reach the level of significance (*p* = 0.11).

## 4. Discussion

The mechanical and thermal experiments conducted in the present study underlined that resins with desktop SLA 3D printers can be potentially used for the fabrication of efficient and durable heat-exchange devices to be used in vascular physiology experiments. Our investigation is essential for basic science, because, to our knowledge, currently no studies are available about the characterization of clear resin material for such research purposes. Our mechanical tests revealed that the studied resin material is sufficiently hard and brite, with a Shore D hardness value of 83.0 ± 0.5. The flexural and tensile parameters of the used resin were relatively low compared to thermoplastics used in additive manufacturing, such as polyamide and PLA, which have significantly higher tensile strength based on a previous study [29]. The results of the mechanical testing were supported by images taken with digital microscopy: the structure of the resin sample showed a glass-like, smooth surface both on the intact and broken surfaces. Thermal conductivity measurements were also performed in order to evaluate if the resin material can be used for the development and fabrication of heat-exchange devices, which can be applied in thermophysiology measurements. According to the thermal analysis of the sample, the heat stability was shown up to slightly above 250 °C, although melting and sample decomposition occurred relatively quickly above that temperature range. These findings confirm that the transparent resin material can be reliably used in biomedical applications. Furthermore, with thermal conductivity measurements, we aimed to examine how the material reacts to the changing temperature conditions. We found that a relatively effective heat insulation can be achieved by using the studied resin material. In addition, we also showed that the application of the fabricated heat-exchange device can maintain a nearly constant temperature in vascular physiology measurements. Based on our initial results, a heat-exchange device was designed and fabricated with SLA printing, and then a series of thermophysiology experiments in rat arteries was carried out by applying the newly developed heat exchanger as an accessory device in a wire myograph system.

By the additive manufacturing of heat-exchange plates, we developed a novel method for the measurement of vasomotor responses in the cold (at temperatures as low as 13 °C). With the cooling plates as accessories of the wire myograph system, our findings can contribute to the ex-vivo study of vasomotor responses of isolated vessels in the cold, which is currently an underrepresented field of research.

Body temperature variabilities influence all biological processes. Living organisms are exposed to different temperatures and dominant animal species on Earth (e.g., reptiles, birds and mammals) are euthermic and maintain their core body temperature in relatively narrow ranges by using behavioral and non-behavioral thermoregulation [30]. In contrast with the temperature of the core, temperatures of the body’s shell (e.g., the skin) vary widely according to the actual environmental temperature [31]. When exposed to cold, the skin temperature of the cutaneous tissue can be close to that of the low ambient temperature due to the vasoconstriction of the cutaneous arteries. If the cold exposure is excessive, then temperature will not only decrease on the surface, but also in the core of the body. Similarly to cold, severe systemic inflammation (e.g., severe sepsis) can be also accompanied by decreased core body temperature (called as hypothermia), which is associated with higher mortality in human patients [32]. It should be also noted that, in contrast with the spontaneously developing hypothermia in the cold or in systemic inflammation, the induction of hypothermia can be used as a therapeutic intervention to improve the outcome of diseases, such as severe traumatic brain injury [33]. Regardless of its etiology, the decreased tissue temperature greatly influences biochemical processes, thereby also vascular reactions.

According to the van’t Hoff law, all biochemical processes speed up 2–3 fold with each 10 °C increase, though this is just a simplification of the phenomenon [34]. The temperature-sensitivity of molecular functions can be expressed with the temperature coefficient Q_10_ that shows the change in the velocity of a chemical reaction caused by 10 °C rise in temperature. Changes in temperature influence the functions of all ion channels usually with Q_10_ of ~2–3; however, there are some channels that can be directly gated by temperature, which are, therefore, characterized by exceptionally high Q_10_ [35]. For example, in case of the heat-sensitive transient receptor potential vanilloid-1 channel, the Q_10_ value is 40 in the temperature range between 41 and 50 °C [35], while, for the cold-sensitive transient receptor potential melastatin-8, the calculated Q_10_ was 24 between 18 and 25 °C [36]. Importantly, both aforementioned temperature-sensitive channels were shown to play important roles in the mediation of vascular responses in the skin [20,37]. With in-silico methods, it is extremely difficult, if not impossible, to predict how temperature-dependent changes in molecular functions would affect physiological responses of different tissues or organs. Hence, it is essential to perform experiments in animal models at different temperatures and study how temperature influences different biological processes. Such studies can help the better understanding of pathological conditions involving temperature changes (e.g., sepsis) and can also help to pave the way towards the development of therapeutic options for safe and controlled modulation of temperature.

As the initial step of translational research with our newly developed heat-exchange device, we tested its application in biomedical research. For that reason, we performed a series of experiments in isolated tail arteries of rats at different temperatures. In our experiments, we demonstrated that the fabricated heat-exchange device could be successfully used as an accessory of the wire myograph system to cool down the experimental chambers and to steadily maintain their temperatures at as low as 13 °C throughout the procedure. We were able to record the vasoconstriction of the arteries in the cold, thereby confirming the viability of the vessels as well as the capability of the system to study vasomotor responses at 13–16 °C. The sympathomimetic drug phenylephrine caused a concentration-dependent vasoconstriction at all studied temperatures (viz., 13, 16, and 36 °C). The extent of phenylephrine’s effect seemed to depend on temperature, as the increase in the isometric force was smaller at all concentrations in the cold (13 or 16 °C) than at 36 °C; however, the difference did not reach the level of statistical significance (*p* = 0.011). We also studied the vasomotor response to KCl, which directly depolarizes smooth muscle cells, thereby leading to vasoconstriction. At the 60 mM concentration, KCl induced vasoconstriction at 36 °C, whereas at 13 and 16 °C, it had no effect. The absence of KCl-induced vasoconstriction was shown previously in arterioles isolated from bigger mammals, such as dogs and pigs; it was suggested to be associated with the temperature-dependence of calcium or potassium homeostasis [38,39,40]; however, the exact mechanisms have remained unknown. Alternatively, the reduced affinity of the Na^+^/K^+^ ATP pump for ATP was shown below 20 °C in cold-sensitive species [41], which could lead to accumulation of sodium within the cells and to a shift of the membrane potential to more positive values, thereby increasing the electromotive force needed for KCl to depolarize the cells. In support of a reduced activity of the Na^+^/K^+^ ATP pump in the cold, when the electrolyte levels were measured in canine arterioles after a 2-h long cold exposure, the intracellular concentrations of sodium and potassium were significantly increased and decreased, respectively [42]. In such a case, increased extracellular KCl concentrations might create enough electromotive force to depolarize the cells. In our experiments, we tested this hypothesis and found that, when we increased the concentration of KCl from 60 to 90 mM, we could observe the KCl-induced vasoconstriction in the cold. These findings demonstrate that our newly developed device is feasible for the investigation of the underlying mechanisms of cold-induced changes in vascular physiology.

As a limitation of using a heat exchanger in wire myography, it should be mentioned that during the experiment the chamber temperature should not be changed because the isometric force-measuring transducers are calibrated to a specific temperature value before the experiment. If the temperature deviates from its value used for calibration, then inaccurate readings and transducer drift (e.g., heat-induced expansion of the electronic parts in the transducer) may occur, introducing large errors into the experiment. Because of this limitation, the system should not be used to study the effects of dynamic temperature variations within the same experiment. It is also important to mention that standard photopolymers for additive manufacturing can have potential cytotoxic effects [43], and the investigated clear resin should not be considered as a non-toxic material. Experiments in cell cultures or living tissues which would require a direct contact with resin are not recommended [43]; however, if the resin is not in direct contact with the biological sample or its bathing solution but only with the experimental device—as was the case in the thermophysiological experiments in the present study—then it can be safely applied in biomedical research.

## 5. Conclusions

The examined transparent resin 3D-printed with desktop SLA device was found as a suitable material for the design, prototyping, and production of heat-exchange devices. The mechanical and thermal stability ensures its potential application in the biomedical field; however, it must be emphasized that further investigations are encouraged in order to find the optimal design and 3D printing parameters for this purpose. The findings of our study shed light on novel ways and methods to fabricate heat exchangers by using a widely available and cost-effective additive manufacturing technology.

When tested in thermophysiology experiments, the 3D-printed heat-exchanger plates as accessories in wire myography allowed us to cool the chambers below the room temperature and to demonstrate temperature-dependent differences in the vasoconstriction of rat tail arteries induced by different substances. To our knowledge, we are the first to report the inhibitory effects of cold on KCl-induced vasoconstriction in isolated rat tail arteries. The combined use of the newly developed cooling device and the rat species establishes a novel, widely accessible, and inexpensive experimental tool to study the effects of cooling on vasomotor responses of mammals. Our findings also suggest that temperature-dependent physiological mechanisms should be widely investigated and better understood in order to discover the background of the thermal changes and their consequences in pathological conditions, as well as in therapeutic implications.

## Figures and Tables

**Table 1:** Printing and post-processing parameters of SLA 3D printing.

	SLA Printing Technology					Post Processing	
Material	Color	Laser Beam Focus Width (µm)	Touchpoint Size (mm)	Raft Type	Layer Thickness (µm)	Washing Time in Isopropyl-Alcohol (min)	Time in 60 °C Ultraviolet Chamber (min)
Clear resin	Clear	85	0.7	Full	100	10	15

**Table 2:** The results of the mechanical characterization of the fabricated heat exchanger.

		Mean	SD
Tensile Young’s modulus	(MPa)	2304.80	42.90
Tensile strength	(MPa)	67.66	1.31
Flexural Young’s modulus	(MPa)	2246.80	66.70
Flexural stress at standard deflection	(MPa)	78.82	1.17
Charpy impact	(kJ/m^2^)	20.70	2.30
Shore D hardness		83.00	0.47

**Table 3:** Corresponding temperature values of the characteristic DSC peaks in the heating cycle of the transparent resin samples (where T_on_ is the initial temperature, T_g_ is the glass transition temperature, and T_end_ is the final temperature of the phase). The data are shown in the mean ± standard error format.

T_on_ (°C)	T_g_ (°C)	T_end_ (°C)	Melting (°C)	Decomposition (°C)
100.82 ± 3.55	122.09 ± 4.15	138.96 ± 1.07	359.11 ± 0.27	438.93 ± 0.38

## Data Availability

Data is contained within the article.
